# Supplementary material for: The Effect of the Addition of Copper Particles in High-Density Recycled Polyethylene Matrices by Extrusion
Source: Polymers (Basel). 2022 Nov 30;14(23):5220. doi: 10.3390/polym14235220 (PMC9739686; doi:10.3390/polym14235220)
Supplement: Supplementary file 1 [file polymers-14-05220-s001.zip › polymers-1991824-supplementary.pdf]

polymers

MDPI

The Effect of the Addition of Copper Particles in High-Density Recycled Polyethylene Matrices by Extrusion

Camila Arcos [1,*], Lisa Muñoz [2], Deborah Cordova [3], Hugo Muñoz [1], Mariana Walter [3], Manuel I. Azócar [3], Ángel Leiva [4], Mamié Sancy [5,6] and Gonzalo Rodríguez-Grau [5,*]

[1] Departamento de Ingeniería Mecánica y Metalúrgica, Facultad de Ingeniería, Pontificia Universidad Católica de Chile, Santiago 7820436, Chile
[2] Instituto de Química, Facultad de Ciencias, Pontificia Universidad Católica de Valparaíso, Valparaíso 2373223, Chile
[3] Departamento de Química de los Materiales, Facultad de Química y Biología, Universidad de Santiago de Chile, Santiago 7820436, Chile
[4] Facultad de Química y Farmacia, Pontificia Universidad Católica de Chile, Santiago 7820436, Chile
[5] Escuela de Construcción Civil, Facultad de Ingeniería, Pontificia Universidad Católica de Chile, Santiago 7820436, Chile
[6] CIEN UC, Pontificia Universidad Católica de Chile, Santiago 7820436, Chile
* Correspondence: cearcos@uc.cl (C.A.); grodriguezg@uc.cl (G.R.-G.)

Table S1. Variation of colonies number and fraction, and viability of *E. coli.*

Sample	Colonies Number	Colonies Fraction	Viability (%)
HDPE	118	112	100
	106		
rHDPE	42	41.5	100
	41		
HDPE Cu-NPs	6	9	8.03
	12		
rHDPE Cu-NPs	50	40	96.3
	30		
HDPE Cu-NCs	1	1.5	1.3
	2		
rHDPE Cu-NCs	17	21	50.6
	25		

Table S2. Variation of colonies number and fraction, and viability of *S. aureus*.

Sample	Colonies Number	Colonies Fraction	Viability (%)
HDPE	>400	400	100
	>400		
	30		
rHDPE	50	50	100
	50		
HDPE Cu-NPs	9	9	2.25
	9		
rHDPE Cu-NPs	49	40.5	81
	32		
HDPE Cu-NCs	2	3	0.75
	4		
rHDPE Cu-NCs	26	39	78
	52		
